# Editorial: Approaches to improve the performance of virtual screening: scoring functions, structural filtration, prediction of physicochemical properties/pharmacological activity

**DOI:** 10.3389/fphar.2025.1658699

**Published:** 2025-08-12

**Authors:** Hélcio Silva Dos Santos, Alexander Veselovsky, Dmitry Nilov

**Affiliations:** ^1^ Center for Exact Sciences and Technology, State University of Vale do Acaraú, Sobral, Ceará, Brazil; ^2^ Graduate Program in Natural Sciences, State University of Ceará, Fortaleza, Ceará, Brazil; ^3^ Graduate Program in Biotechnology, Northeast Network of Biotechnology, State University of Ceará, Campus Itaperi, Fortaleza, Ceará, Brazil; ^4^ Institute of Biomedical Chemistry, Moscow, Russia; ^5^ Belozersky Institute of Physicochemical Biology, Lomonosov Moscow State University, Moscow, Russia

**Keywords:** virtual screening, drug design, data sets, machine learning, deep learning, molecular docking, molecular dynamics, generative models

## Introduction

Virtual screening (VS) has become a widely used tool in drug discovery, enabling the identification of potential drug candidates from large compound libraries. However, the success of VS heavily relies on the accuracy and efficiency of the approaches used. There are several challenges that need to be addressed to improve the performance of VS, including (Muegge et al., 2024):

1. *Scoring functions*: Scoring functions, mathematical algorithms predicting ligand-protein binding affinity, remain imperfect with limitations in accuracy and high false positive rates.
2. *Structural filtration*: Structural filtration removes compounds with unfavorable structures for target binding, such as those that are too large, too small, contain undesirable functional groups, or do not form the required interactions with a protein target.
3. *Prediction of physicochemical properties/pharmacological activity*: Beyond binding affinity, predicting the physicochemical and pharmacological properties of VS-identified compounds, such as solubility, permeability, metabolism, and toxicity, is crucial.
4. *Management of large datasets*: Virtual screening involves screening vast compound libraries, potentially containing millions or billions of compounds, which poses significant computational challenges for data management and analysis.
5. *Experimental validation*: Experimental validation of virtual screening results, while crucial for confirming binding affinity and activity, is often expensive and time-consuming. Therefore, developing more efficient validation methods remains an important challenge.

Overcoming these challenges is essential to improving the performance of virtual screening and accelerating the discovery of new drugs (de Oliveira et al., 2023). This Research Topic showcases recent advancements and innovative strategies for refining virtual screening effectiveness in drug discovery. It comprises seven original research papers describing advanced docking and post-docking approaches (DFT, molecular dynamics, and MM-PBSA, etc.) for effectively selecting active ligands with desired properties.

The challenges and possible solutions mentioned above can be verified in the study conducted by Elsaman et al. that demonstrates a computational screen of 460,000 compounds from the National Cancer Institute library to identified KHK-C inhibitors using pharmacophore-based virtual screening, a multi-step approach prioritized compounds with strong binding affinity, favorable pharmacokinetic profiles, and high efficacy to find inhibitors of ketohexokinase C (KHK-C) a key enzyme in fructose metabolism, a promising therapeutic strategy to combat diseases like obesity and diabetes caused by high fructose intake. This efficient, cost-effective *in silico* approach selected the compound 2 as a promising KHK-C inhibitor with favorable predicted pharmacokinetics and toxicity, suggesting its potential as a treatment for fructose-driven metabolic disorders and meriting further study.

Another approach driven by Zhang et al. underscores the importance of natural product chiisanoside isolated from *Acanthopanax sessiliflorus* as valuable source for developing targeted therapies against cisplatin-induced ototoxicity hearing loss, a widespread issue affecting communication and quality of life over 5% of the global population. *A*. *sessiliflorus*, consumed for over a century in some regions, has shown potential against cisplatin-induced ototoxicity (CIO). This study screened 26 chiisanoside derivatives, and found that compound 19 significantly protected against CIO damage. In this way, using pharmacophore-based virtual screening, researchers discovered that compound 19 can prevent ototoxicity, finding a novel approach for treating hearing loss.

The research carried out by Xu et al. based on molecular docking and molecular dynamics simulations was performed to evaluate the binding stability between 23 compounds each demonstrating significant inhibitory effects on MDA-MB and MCF-7 breast cancer cell lines a significant global health issue influenced by a combination of genetic predispositions and lifestyle factors and the human adenosine A1 receptor-Gi2 protein complex (PDB ID: 7LD3). The study demonstrated that compound 5 has stable binding to the adenosine A1 receptor and compounds 6–9 has strong binding affinities, which guided the design and synthesis of molecule 10 with potent antitumor effects on MCF-7 cells. This research deepens our understanding of molecular interactions by combining reverse drug screening, molecular modeling, and *in vitro* validation, it lays a solid foundation for future drug discovery efforts in breast cancer treatment.

Shahwan et al. showed that a virtual screening of 3,648 drug molecules targeting dysregulation of monoamine neurotransmitters (MAO-B) binding pocket identified the compounds brexpiprazole and trifluperidol as promising candidates due to their high binding potential and favorable drug profiles to combat depression and parkinson’s disease (PD) two major psychiatric and neurological challenges that affecting millions and burdening healthcare systems. All-atom molecular dynamics simulations (300 ns) of MAO-B complexes with these ligands revealed minimal structural changes in MAO-B and significant stabilization throughout the simulation. These findings suggest that brexpiprazole and trifluperidol are potential MAO-B inhibitors that warrant further experimental investigation for targeted therapies for depression and Parkinson’s disease.

Since the emergence of SARS-CoV-2, highly transmissible variants have driven interest in drug repurposing, particularly antimalarials. For this purpose, Quijada et al. conducted *in vitro* assays in two host mammalian cell systems, Vero-E6 and Calu-3 cells to assess the antiviral activity of the 26 antimalarial and antiviral compounds against the Delta and A2.5 variants isolated in Panama (2020–2022). In Vero-E6 cells, chloroquine significantly inhibited the Delta variant, while amodiaquine, artemisone, and ivermectin were active against the A2.5 variant. In Calu-3 cells, chloroquine, amodiaquine, artesunate, lumefantrine, and hydroxychloroquine were effective against the Delta variant, whereas only amodiaquine and arteether showed activity against the A2.5 variant, demonstrating variant- and cell-type-dependent responses. This study highlights the importance of choosing relevant cell models for SARS-CoV-2 research, as drug efficacy differs based on viral variant and host cell type.

Malaria, caused by Plasmodium parasites and transmitted by Anopheles mosquitoes, remains a significant global health threat. Despite continuous efforts to develop safer and more effective medications, the disease poses major challenges for new drug discovery. The most dangerous species, *P. falciparum*, degrades hemoglobin and is developing increasing drug resistance, highlighting the urgent need for new therapeutic targets. Aspartyl proteases like plasmepsin X, which is crucial for the parasite’s survival by digesting hemoglobin, are promising targets for new antimalarial drugs. A recent study by Almuqdadi et al. used a fragment-based virtual screening approach to identify potential inhibitors. Starting with a library of over 14,000 compounds, they systematically narrowed the candidates down to 20 priority compounds that with ran molecular dynamics (MD) simulations identified compounds 3 and 4 as superior inhibitors, offering new therapeutic possibilities against drug-resistant malaria. Ultimately, this work contributes new insights and therapeutic possibilities in the ongoing battle against drug-resistant malaria.

Computational assay was used for identifying millet-derived compounds that antagonize the interaction between bisphenols and estrogen-related receptor gamma. The exposure of bisphenol A and its analogs on humans through oral, transdermal, and respiratory routes is associated with reproductive, developmental, metabolic, and carcinogenic disorders. However, they are widely used in industries, and compounds capable of neutralizing their toxic effects may be of interest. In the study developed by Pathak and Kim 59 millet phytochemicals were virtually screened via molecular docking, prioritizing the top ten based on ADMET profiles, and the top five compounds were further analyzed using DFT, molecular dynamics, and MM-PBSA. It allowed elucidation of the mechanisms by which natural compounds antagonize interactions of bisphenol with estrogen-related receptor gamma and identification of key receptor residues. The selected compounds were predicted to be competitive inhibitors and could be potentially used in the development of future therapeutics or food supplements.

To guide future research, key challenges remain: integrating diverse data types into virtual screening, developing innovative scoring functions for complex targets, and creating robust structural filters that account for protein flexibility. Addressing these questions, along with improving the prediction of physicochemical properties and pharmacological activity, is crucial. The diverse approaches presented in this Research Topic offer a strong foundation and may stimulate future research, ultimately leading to the discovery of more effective and safer drugs.
